# Mental Nursing

**Published:** 1919-04-12

**Authors:** 


### THE LIFE OF A NURSE: ITS MORAL AND MATERIAL ADVANTAGES.

### IV.—Mental Nursing.

MENTAL nursing is an absorbingly interesting branch of nursing for those who will enter it from the right motive, to alleviate the sufferings of a class who, till recent years, have been greatly misunderstood, suspected and shunned. Institutions for the treatment of these patients are termed Mental Hospitals, for their inmates are mentally ill, and sometimes physically so also.

### THE SUCCESSFUL MENTAL NURSE.

The treatment consists largely in educating the patients to behave like normal beings, to win back their self-respect, to take a more hopeful outlook on life and to occupy themselves usefully in some work such as needlework, cooking, or laundry. The most successful nurse will be one who is sympathetic with her charges, who will enter into their troubles, and be ever ready to help each one individually and let them feel she is one to whom they can turn for help. She must be observant to notice any change in their mental condition which may denote coming danger, and also be able to detect symptoms of physical illness which are often obscured by their mental condition. She must also be conscientious in obeying the rules of the hospital, which are made for the safeguarding of patients and staff. She should be good tempered, and if she is able to see the lighter side of life, it is certainly a great advantage.

Many mental patients are only insane on one subject, and are good companions in other respects. It is a popular idea that they must all be violent, and therefore not fit for young girls to nurse; but this is quite a fallacy. Some, of course, are, and the nurse who is most successful in controlling them is one who has learnt to control herself and has a genuine pity for, and interest in, those who are mentally not strong enough to control themselves. Should she feel fear, she must endeavour not to show it. Of all the branches of nursing, I think mental nursing is the one that calls for the highest qualifications in character.

There are many things to be done for these patients which a nurse might be tempted to call "menial," but if she thinks of the patients as children, which in mind they really are, she will soon realise it is her privilege to serve them as need arises. There is drudgery in all professions, but when it is borne for those who cannot help themselves, it is a noble form of self-sacrifice.

### SOME ATTRACTIONS.

Among the attractions to this vocation are :—(i.) That it is easy to enter without a long time of waiting, as at present it is not so well understood and sought after as it should be. (ii.) The hospitals are usually situated in the country or, at any rate, in large grounds, supplying their own farm produce, fruit, vegetables, flowers, home-made bread, etc. (iii.) The off-duty time is good and the quarters comfortable. (iv.) There are weekly concerts, entertainments or dances in the winter, and outings or picnics in the summer for the patients, in which the staff join, as well as staff dances. (v.) Lectures are given regularly by the medical officers and examinations held in first aid and home nursing and the preliminary and final examination for the certificate of the Medico-Psychological Association. This can be obtained after three years' training, and with it usually a rise is given in salary. If the nurse has already a certificate of three years' general training, she is excused one year of the mental course and the preliminary examination. (vi.) The salary is good, usually starting for probationers at £33 10s. per annum, with board, residence, laundry, and uniform (rising annually). A small deduction is made towards a pension. Medical attendance is provided in case of illness. Promotion is fairly rapid; but there is a general tendency now to appoint matrons who have both general and mental training. (vii.) Mental nursing under public bodies is a pension service. The amount of pension depending upon the length of service and the rate of salary when retiring.

To any girls who feel drawn to the life of a mental nurse and think they have the necessary qualifications for success, I would say "Try it, and don't be deterred because others throw cold water on your aims." The London County Council has large mental hospitals near London, and each county has one or more such institutions. Application should be made to the matron of the hospital selected, who will send an application form, which must be returned duly filled in, and the candidate if accepted will have to pass a medical examination upon arrival at the hospital.

☞ Previous articles appeared on February 8 and 15, and March 8, p. 503.

